# Prevalence of multidrug-resistant *Escherichia coli* isolates and virulence gene expression in poultry farms in Jos, Nigeria

**DOI:** 10.3389/fmicb.2024.1298582

**Published:** 2024-06-12

**Authors:** Ebere Roseann Agusi, Dennis Kabantiyok, Nicodemus Mkpuma, Rebecca Bitiyong Atai, Chidinma Okongwu-Ejike, Ebun Lydia Bakare, James Budaye, Kabiru Garba Sule, Rindah Joy Rindaps, Gyallak Kingsley James, Benshak John Audu, Godwin Ojonugwa Agada, Oyelola Adegboye, Clement Adebajo Meseko

**Affiliations:** ^1^Biotechnology Centre, National Veterinary Research Institute, Jos, Nigeria; ^2^Fleming Laboratory Microbiology Department, National Veterinary Research Institute, Jos, Nigeria; ^3^Menzies School of Public Health, Charles Darwin University, Darwin, NT, Australia; ^4^Regional Laboratory for Animal Influenza & Transboundary Animal Diseases, National Veterinary Research Institute, Jos, Nigeria

**Keywords:** One Health, biosecurity, multidrug resistance, virulence genes, livestock, *Escherichia coli*

## Abstract

**Introduction:**

Antimicrobial resistance is increasingly becoming a global health concern. This study aimed to investigate and report MDR *Escherichia coli (E. coli)* prevalence, resistance, and virulence genes from poultry in Jos, Plateau State, Nigeria.

**Methods:**

The samples were analyzed using microbiological standard methods and polymerase chain reactions (PCRs).

**Results:**

A total of 179 cloacal swabs were collected from bothlocal and exotic poultry breeds, of which 99.4% (178/179) tested positive for *E. coli*. Among these culturally identified samples, 99.4% (177/178) were furtherconfirmed *Escherichia coli* with a molecular weight of 401 bp. Multidrugresistance of 45% (80/178) was observed from the confirmed isolates. PCR assays were conducted to detect genes associated with resistance to antibiotics, specifically, tetracycline (*tetA* gene), sulfonamide (*sul1* gene), ampicillin (*ampC* gene), and quinolone (*gyrA* gene). Antimicrobial susceptibility test (AST) results revealed substantial antibiotic resistance, with 81.9% (145/177) of the isolates being resistant to tetracycline, 80.2% (142/177) to quinolone, 69.5% (123/177) to sulfonamide, and 66.1% (117/177) to ampicillin. Further analysis on 18 isolates that showed resistance to up to four different antibiotics was carried out using multiplex PCR to detect *eae, hlyA, rfbE, fliC,* and *fstx* virulence genes. The study found that 44.4% (15/18) of the isolates were positive for the *eae* gene, 27.7% (5/18) for *stx*, 22.2% (4/18) for *rfbe* gene, and 5.5% (1) for *hlya* gene, and none tested positive for *fliC* gene.

**Conclusion:**

These results showed high antibiotic resistance, virulent genes, and significant levels of MDR in *E. coli* from poultry. This study highlights the urgent need for antimicrobial stewardship practices within the poultry industry due to their profound implications for food safety and public health. This issue is particularly critical in Nigeria, where poultry farming constitutes a significant portion of smallholder farming practices.

## Highlights

What is already known about this topic

- Antimicrobial resistance is a growing concern for One Health, especially in the livestock industry.
- Poultry farms close to humans pose a risk of transmission episodes of zoonotic MDRs
- Up to 70% of antibiotics are excreted by animals as unmetabolized substances, which means an increase in selective pressure leading to environmental antimicrobial resistance.

What this study adds

- The presence of resistant genes in free-range poultry in the communities suggests environmental contamination by antimicrobial genes.
- There is a high prevalence of drug-resistant *Escherichia coli* in poultry from Jos.

## Introduction

Multidrug resistance (MDR), a phenomenon often used to describe bacteria that are resistant to three or more antibiotic classes, has become increasingly common (Kallau et al., 2018). Recently, antimicrobial resistance (AMR) has gained global recognition due to the rise in multidrug-resistant organisms such as *Escherichia coli* (*E. coli*), which has led to increased economic burden and mortality, especially in Nigeria. Aworh et al. (2019) posited that MDR among many organisms has become a huge challenge to infectious disease management, and it is increasingly being reported in bacteria, often mediated by genetic mobile elements, such as integrons, transposons, and plasmids (Nnaji et al., 2021).

*E. coli* strains that are multidrug-resistant from animal and human isolates are ubiquitous in different parts of the world (O’Neill, 2019), with the non-pathogenic strains having the likelihood of being an important reservoir of resistance genes. Extensive usage of antibiotics in livestock production for disease prevention (prophylaxis and metaphylaxis), treatment, and growth promotion is one of the principal factors in the spread of antibiotic resistance (Brower et al., 2017; Hassan et al., 2021). Antimicrobials are routinely added to animal feeds, and bacterial populations are repeatedly exposed to sub-therapeutic doses required for the development and spread of MDR (Brower et al., 2017). In addition, in many developing countries in Asia and Africa, there is little or no restriction on antibiotic usage, and antimicrobial agents are less restricted in local drug stores without prescription. Such practice has led to the misuse of antibiotics, leading to resistance among isolates from animals, food sources, and the environment (Agada et al., 2014).

*E. coli* is one of the commensal bacteria found in the gastrointestinal tracts of humans, poultry, and mammals (Akond, et al., 2009; Olatoye et al., 2012) but may become opportunistic, leading to disease states (Akond, et al., 2009). Although most *E. coli* strains are commensal, a few highly pathogenic ones carry *stx2* and *eae* genes, causing watery and bloody diarrhea associated with life-threatening diseases, such as thrombocytopenic purpura, hemolytic uremic syndrome, hemorrhagic colitis (Al Azad et al., 2019), and extra-intestinal diseases. This bacterium can be acquired through contaminated water, soil, and food (Okorie-Kanu et al., 2016), with most human infections occurring from contaminated food products of poultry origin (Hamisi et al., 2014). Globally, *E. coli* effects on poultry production can be severe (Hamisi et al., 2014), and it is a public health threat in sub-Saharan African countries, including Nigeria. Some drug-resistant *E. coli* strains, which may not directly cause disease, remain significant in public health as a reservoir of drug-resistant genes that can be transferred to humans (Aworh et al., 2019).

Poultry is a good source of high-quality animal protein for the growing population of Nigeria and a vital part of the Nigerian economy, which provides income and livelihood for smallholder farmers (Awoyomi et al., 2022). It accounts for approximately 25% of local meat from livestock produced in Nigeria (Anosike et al., 2018). Due to the increased consumption of poultry products in Nigeria, there is dependence on poultry production by many families for income and livelihood (Agada et al., 2014; Aworh et al., 2019), as well as antimicrobial agents to boost production by minimizing diseases. These antimicrobial agents are used for prophylaxis, treatment, and growth promotion (Brower et al., 2017; Hassan et al., 2021). The addition of these antimicrobial agents poses a significant risk to the emergence of some resistant bacteria either through genetic or non-genetic means, thereby making poultry a major reservoir of antimicrobial-resistant *E. coli* (Okorie-Kanu et al., 2016).

Although poultry may serve as a significant reservoir of resistant bacteria in food animals, there is a paucity of information on the multidrug resistance pattern of *E. coli* in poultry in the study area. This study was designed to detect *E. coli* from local and exotic poultry breeds in Jos, Plateau State, and to examine their antimicrobial susceptibility pattern and the presence of multidrug-resistant and virulence genes from the isolated bacteria.

## Materials and methods

### Data collection

Between November 2019 and February 2020, cloacal swabs were collected from birds in 26 randomly selected farms in Jos South Local Government Area, Plateau State, Nigeria. Birds were grouped into two categories: caged (intensively managed) and free-ranged (extensively managed) local birds. Additionally, farm-level data such as biosecurity, vaccination status, source of water, previous use of antibiotics, and farm management (intensive or extensive) were obtained using interviewer-administered questionnaires.

### Sample size determination

The sample size for the study was calculated based on the prevalence of *E. coli*, aiming for a 95% confidence level and a 5% margin of error. Utilizing the formula, N=Z^2×P(1−P) /L^2, where N represents the required sample size, Z is the Z-statistic corresponding to a 95% confidence level (1.96 for two-tailed tests), P is the prevalence of *E. coli*, and L is the allowable error margin at 5%. For this study, P was taken as 13.4% based on previous findings by Shecho et al. (2017). Substituting these values into the formula, we computed N as (1.96^2*0.134*0.866)/(0.05^2) which resulted in approximately 178. To accommodate the study design and ensure robustness, the sample size was rounded up to 180 cloacal swabs.

### Isolation of *E. coli*

Samples were inoculated into bacteriological peptone water and incubated for 24 h at 37 ± 0.5°C before streaked onto MacConkey agar. *E. coli* was isolated as Gram-negative short rods from lactose-fermenting colonies that were positive for indole produced acid and gas in the triple sugar iron agar test but negative for citrate and urea. To further confirm isolates, they were cultured on eosin methylene blue agar (EMB) (FLUMEDIA, United Kingdom), which is a selective medium for *E. coli*. Only colonies that produced the green metallic sheen on EMB were used for the antimicrobial susceptibility test. The isolated *E. coli* were subjected to molecular confirmation using *E. coli* genus-specific primer targeting the 16S rRNA (Sabat et al., 2000).

### Antimicrobial susceptibility testing

The agar disk diffusion method was used to determine resistant, intermediate, or susceptible isolates to the antibiotics. A total of seven antibiotics spanning across five classes of antibiotics were used (cefoxitin 30 μg, oxacillin 10 μg, erythromycin 10 μg, sulfamethoxazole/trimethoprim 1.25/23.7 μg, nalidixic 30 μg acid, ampicillin 10 μg, and ciprofloxacin 5 μg). A turbidity of 0.5 McFarland standard of the isolate was streaked onto a Mueller–Hinton agar (HiMEDIA, Bombay, India) plate before the respective antibiotic disks were placed aseptically on the surface. Zone clearing was measured in millimeters and recorded as resistance, intermediate, or susceptible following CLSI standards. Isolates that showed resistance to at least three different classes of antibiotics will be classified as MDR *E.coli*.

### PCR confirmation of isolates and detection of virulence and antimicrobial genes

We conducted a microbiological standard culture of cloacal swab samples collected from both exotic breeds and local birds. Subsequently, samples that showed positive indications for *E. coli* were further analyzed through molecular techniques using a 16S rRNA primer. To identify antibiotic resistance genes, we employed a selection process for PCR analysis based on the outcomes of antibiotic susceptibility testing methods. This approach aimed to ascertain the presence of resistance genes to various antibiotics, such as quinolone, tetracycline, ampicillin, and sulfonamide, each identified by their distinct molecular weights. Amplicons were separated on a 1.5% agarose gel at 80 V for 40 min.

To dedect the virulence genes in the isolates, we applied the method previously described by Fusaro et al. (2009) and utilized primer as per the guidelines of Wang et al. (2002). The reaction conditions were first optimized to obtain optimum reaction and temperature conditions. After optimization, *eae, hlyA, rfbE, fliC,* and *stx2* genes for virulence genes were performed using multiplex PCR.

### Ethical clearance

All procedures involving animals in the study were conducted following guidelines obtained from the ethics committee of the National Veterinary Research Institute NVRI, Vom. Approval reference number AEC/03/76/19.

### Data analysis

Descriptive statistics such as frequencies and percentages were used to describe the data. Group frequencies were compared using the chi-squared or Fisher’s exact test where applicable. Poisson regression was utilized to assess the association between farm-level characteristics and the presence of *E. coli* and MDR, while Firth’s logistic regression model that corrects for bias in rare events was used to investigate the effect of farm-level factors on the presence of pathogenic isolates (Beaujean and Grant, 2016; Puhr et al., 2021).

## Results

### Isolation and prevalence of *E. coli*

**Figure 1:**
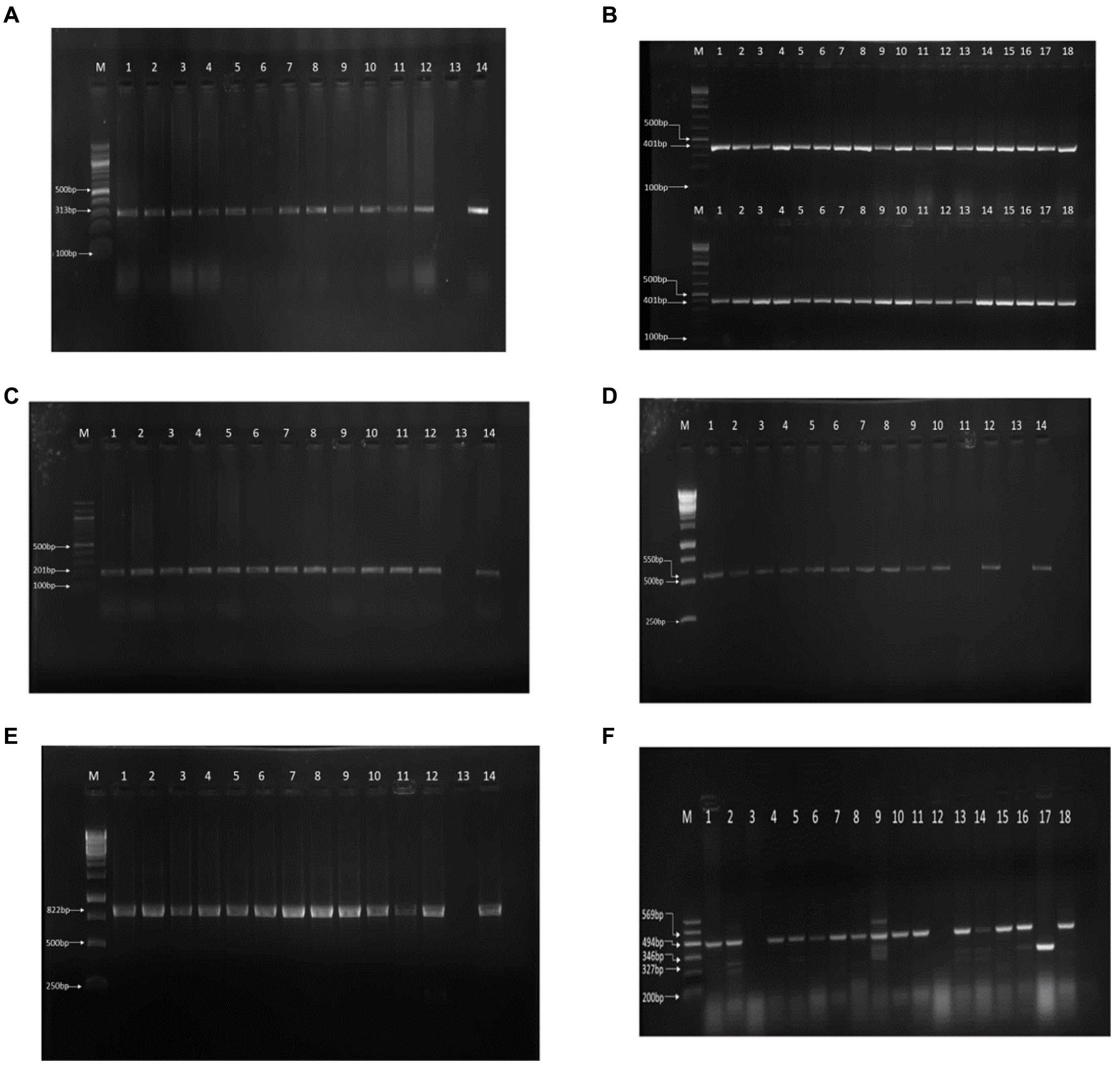
PCR results on gel electrophoresis. **(A)** 16S rRNA PCR result. **(B)** *gyrA*-resistant gene. **(C)** *tetA*-resistant gene. **(D)** *ampC* resistant gene PCR resistant gene PCR result. **(E)** *sul1* resistant gene PCR result. **(F)** Multiplex PCR of the virulence genes.

Of the 179 cloacal swab samples from exotic breeds and local birds from 26 farms subjected to microbiological standard culture, 178 (99.44%) were positive for *E. coli* (Table 1). Molecular analysis confirmed 177 of the samples to be positive for *E. coli* with a molecular weight of 401 bp. In total, 36 isolates that showed a strong bright band (Figure 1A) were further screened.

**Table 1:** Association between farm-level characteristics and prevalence of *E. coli* and MDR.

Factors	*E.coli* IRR (95% CI)			MDR IRR (95% CI)		
	n/N(%)	Univariate	Multivariable	n/N(%)	Univariate	Multivariable
*Overall*	178/179 (99.4)			80/178 (45.0)		
Previous use of antibiotics						
Yes	120 (71.0)	0.29 (0.21, 0.41)***	0.42 (0.18, 1.07)	67 (89.3)	0.99 (0.50, 2.23)	3.14 (0.57, 13.2)
No	49 (79.0)			8 (10.7)		
Vaccination						
Yes	108 (70.0)	0.25 (0.17, 0.39)***	2.16 (0.80, 5.60)	51 (85.0)	0.35 (0.18, 0.77)**	0.64 (0.19, 3.31)
No	27 (70.0)			9 (15.0)		
Source of water						
Borehole	116 (77.4)	6.26 (4.58, 8.67)***	7.08 (4.64, 10.7)***	64 (81.0)	12.9 (7.57, 23.5)***	21.3 (11.2, 42.0)***
Well	56 (72.6)			15 (19.0)		
Farm management						
Extensive	40 (73.3)	3.69 (2.56, 5.20)***	NAN	9 (11.4)	1.56 (0.73, 2.96)	NAN
Intensive	132 (76.7)			70 (88.6)		
Biosecurity						
Yes	24 (76.7)	0.51 (0.32, 0.77)*	1.06 (0.65, 1.68)	17 (32.1)	1.20 (0.66,2.11)	2.82 (1.46, 5.29)**
No	120 (73.3)			36 (67.9)		

^***^ *p* < 0.0001, ^**^ *p* < 0.001, ^*^ *p* < 0.05.

### Antimicrobial susceptibility

We reported resistance to more than 3 classes of antibiotics in 80 out of 178 cases (45.0%) and found at least 1 MDR isolate in all poultry farms sampled. Further exploration into antibiotic resistance revealed significant findings. PCR analysis, informed by antibiotic susceptibility testing, indicated that 80.23% (142 out of 177) of the samples were resistant to quinolone (313 bp), 81.92% (145 out of 177) to tetracycline (201 bp), 66.10% (117 out of 177) to ampicillin (550 bp), and 69.50% (123 out of 177) to sulfonamide (822 bp), as detailed in Figures 1B–E. Consequently, 18 isolates, which were positive for all four antibiotic resistance genes, were selected for more in-depth analysis. The study also employed optimized multiplex PCR for the detection of virulence genes, revealing the presence of the *eae* gene (494 bp) in 8 out of 18 analyzed isolates, the *hlyA* gene in 1 isolate, *the rfbE* gene in 4 isolates, and *stx2* gene in 5 isolates, with the *fliC* gene being absent in all tested isolates.

Amplicons were analyzed using agarose gel electrophoresis to separate DNA fragments based on size. A 1.5% agarose gel was utilized, and the electrophoresis was run at 80 V for 40 min. In the analysis of the 16S rRNA gene, lanes 1 through 18 contained isolates with a 401-bp fragment, and lane (M) served as the molecular size marker, indicated by a 100-bp ladder, to facilitate the estimation of DNA fragment sizes (Figure 1A). Similarly, for the detection of the *gyrA* resistance gene, lanes 1 to 12 harbored isolates that contained the *gyrA* gene, characterized by a 313-bp fragment. Lane 13 was designated as the negative control (containing no DNA), and lane 14 was the positive control, with a known positive isolate exhibiting the same 313-bp *gyrA* gene fragment (Figure 1B). The analysis for the *tetA* resistance gene followed the same procedure, where lanes 1 to 12 contained isolates with the *tetA* gene (201 bp), and lanes 13 and 14 served as the negative and positive controls, respectively, mirroring the setup for *gyrA* (Figure 1C).

Furthermore, multiplex PCR targeting various virulence genes revealed that among the isolates analyzed, 8 harbored the *eae* gene (494-bp), 1 contained the *hlyA* gene (569-bp), 4 possessed the *rfbe* gene (327-bp), and 5 had the *stx2* gene (346-bp), as separated on a 1.5% agarose gel run at 80 V for 40 min. The molecular marker lane (M) again featured a 100-bp ladder to assist in the sizing of these amplicons (Figure 1F). This comprehensive analysis provided a detailed overview of the presence and distribution of resistance and virulence genes among the isolates, leveraging both single and multiplex PCR approaches for the identification of specific gene fragments indicative of bacterial resistance and virulence.

### Association between farm-level risk factors and the presence of *E. coli* and MDR

Table 1 represents farm-level data and its association with the prevalence of *E. coli* and MDR, while Table 2 presents the association between various farm-level characteristics and the presence of pathogenic isolates across 26 farms. In the univariate analysis, we found a significant reduction in *E. coli* among farms that previously used antibiotics, with an IRR of 0.29 (95% CI: 0.21, 0.41). However, this association was not statistically significant in the multivariable model after adjusting for other farm factors (IRR: 0.42, 95% CI: 0.18, 1.07).

**Table 2:** Association between farm-level characteristics and the presence of pathogenic isolates.

Factors	Number of farms	Presence of pathogenic isolates, OR (95% CI)
*Overall*	26	4/26 (15.4)
Previous use of antibiotics		
Yes	15/25 (60.0)	5.88 (0.49, 826)
No	10/25 (40.0)	
Vaccination		
Yes	13/26 (50.0)	5 (0.39, 711)
No	13/26 (50.0)	
Source of water		
Borehole	4/25 (16.0)	0.44 (0.05, 5.56)
Well	21/25 (84.0)	
Farm management		
Intensive	16/25 (64.0)	0.15 (0.00, 1.64)
Extensive	9/25 (36.0)	
Biosecurity		
Yes	3/22 (13.6)	19.4 (1.62, 394)^*^
No	19/22 (86.4)	

Multivariate analysis is not feasible due to sample size. ^*^ *p* < 0.05.

Similarly, vaccination was initially associated with a lower prevalence of *E. coli* (IRR: 0.25, 95% CI: 0.17, 0.39) in univariate analysis, but the significance diminished in the multivariable context (IRR: 2.16, 95% CI: 0.80, 5.60). Borehole water usage was strongly associated with higher *E. coli* presence (IRR: 6.26, 95% CI: 4.58, 8.67) in the univariate model, an association that remained robust and even more pronounced in the multivariable analysis (IRR: 7.08, 95% CI: 4.64, 10.7), underscoring borehole water as a critical factor for bacterial contamination.

The analysis of MDR prevalence also yielded similar significant results. The source of water again stood out, with borehole water associated with a substantially higher prevalence of MDR organisms (univariate IRR: 12.9, 95% CI: 7.57, 23.5), which further increased in the multivariable model (IRR: 21.3, 95% CI: 11.2, 42.0). On the other hand, biosecurity measures showed a protective effect against MDR in the multivariable model (IRR: 2.82, 95% CI: 1.46, 5.29), suggesting effective biosecurity practices that can significantly reduce the prevalence of MDR bacteria despite the univariate model showing a less pronounced association (IRR: 1.20, 95% CI: 0.66, 2.11).

Table 2 examines the association between various farm-level characteristics and the presence of pathogenic isolates across 26 farms. The overall prevalence of pathogenic isolates is reported as 15.4%. Although most of the farm-level factors (except biosecurity measures) were not significantly associated with the presence of pathogenic isolates, they showed interesting associations. For example, farms with a history of antibiotic use exhibited higher odds, farms using borehole water had lower odds, and intensive farm management practices were associated with notably lower odds. In contrast, farms implementing biosecurity practices had significantly higher odds of pathogen presence (OR = 19.4; CI: 1.62 to 394). This counterintuitive finding could reflect complexities not captured by the data, such as the possibility that farms with higher risk profiles or prior pathogen issues are more likely to implement biosecurity measures.

### Virulence detection and antimicrobial genes

Table 3 presents the summary of the PCR gel and multiplex PCR of the virulent genes. For single PCR, the highest detection rate was observed for the 16SrRNA gene, with 177 (99.4%) positive cases. Other genes detected with varying frequencies include *gyrA* (19.7%), *tetA* (18.0%), *ampC* (33.7%), and *sull* (30.3%), reflecting a diverse presence of virulence factors. In contrast, multiplex PCR, which allows for the simultaneous detection of multiple genes, showed a considerably lower detection rate across different genes, with *eae* detected in 8 cases (4.5%), *stx* in 5 cases (2.8%), and *rfbe* in 4 cases (2.2%). Notably, *fliC* was not detected in any samples (0.0%), and *hyla* was found in only 1 case (0.6%).

**Table 3:** Summary of single PCR and multiplex PCR detection for antibiotic and virulence genes.

Target gene	PCR result (No of Positive)		Target gene	Multiplex PCR (No of Positive)	
	*n*	%		*n*	%
16SrRNA	177	99.4	*eaE*	8	44.4
*gyrA*	35	19.7	*fliC*	0	0.0
*tetA*	32	18.0	*stx*	5	27.7
*ampC*	60	33.7	*hylA*	1	5.5
*sull*	54	30.3	*rfbE*	4	22.2

## Discussion

In this study, a comprehensive analysis was conducted on 179 cloacal swab samples from both exotic breeds and local birds to investigate the presence of *E. coli* and associated antibiotic resistance in farms in Jos Plateau, Nigeria. Microbiological standard culture techniques initially identified 178 (99.4%) samples as positive for *E. coli*, which was further confirmed through molecular analysis using a 16S rRNA primer for 177 samples, each exhibiting a molecular weight of 401 bp. Among these, 36 isolates were particularly notable for displaying very strong bright bands. The high occurrence of the positive *E. coli* isolates by PCR from this study supports the findings of Al Azad et al. (2019). As a ubiquitous microbe often isolated from the gastrointestinal tract of warm-blooded animals (Desvaux et al., 2020), its highly promiscuous nature facilitates its acquisition of antimicrobial resistomes (Nhung et al., 2016), thereby making it a tool for the spread of antimicrobial-resistant genes in the food chain and the environment. Against this backdrop, a high prevalence (99.44%) of *E. coli* may have deleterious consequences for both human and animal health (Kwoji et al., 2019). Consequently, infections caused by resistant pathogenic *E. coli* depict high morbidity and negative economic downturn for livelihood and poultry businesses. Environmental contamination by antibiotic residue and AMR genes are a major driver of AMR in livestock because up to 70% of antimicrobials administered to livestock have been reported to be released as unmetabolized agents (Bhushan et al., 2017).

At least one MDR isolate was reported in all poultry farms sampled, which is consistent with the study by Adebowale et al. (2022), which showed a lesser MDR value of 56.3%. The higher prevalence of MDR in this study may be connected to poor biosecurity on farms and the excessive use of antibiotics by farmers. The resistance to more than three classes of antibiotics is indicative of extensive antimicrobial usage. A study by Enany et al. (2019) showed the connection between the frequent use of antibiotics in farm production and treatment and the emergence of multidrug resistance in different bacterial pathogens. Similarly, other studies have emphasized the rising trend in AMR and MDR in low- and middle-income countries such as Nigeria (Osman et al., 2018; Kabantiyok et al., 2023).

What are the implications of a high MDR ubiquitous microbe on the environment, humans, and food value chain? First, there have been reports of AMR and antimicrobial-resistant genes (ARGs) from the environment around animal farms, including poultry and other biological spaces (Mazhar et al., 2021; Mkpuma et al., 2021) supported by the isolation of similar antibiotic-resistant genes simultaneously from human and poultry samples (Johnson et al., 2007). Apart from prophylactic administration, poultry farming benefits from the use of heavy metals in animal feed as growth promoters and has served as a tool for selective pressure in the emergence and maintenance of ARGs in the environment (Muhammad et al., 2020). Furthermore, Manyi-Loh et al. (2018) suggested that the inclusion of antimicrobial agents in feed production as growth promoters by farmers to meet the high demand for animal protein in developing countries may also cause bacteria to develop resistance to the antibiotics used. The excessive use of antibiotics and heavy metals expose humans to a cocktail of antimicrobial-resistant zoonosis, which is a fall-out of selective pressure from unmetabolized residues of antibiotics and heavy metals in the environment. Second, this leads to acquired resistance to newly developed and last-resort antibiotics, which presents a challenge for humans and animals, especially in the management of health outcomes. The emergence of resistance limits the choice of drugs and makes treatment more expensive. In a recent report by Laxminarayan (2022), the estimated number of deaths in 2019 due to AMR was 1.27 million, a figure greater than deaths due to malaria (627,000) and HIV (680,000) put together and an astounding 4.95 million deaths due to AMR-related complications. Antimicrobial resistance is seen to be a concern for the economy as it threatens food security, animal welfare, longer treatment cycles, and public health worldwide (Selaledi et al., 2020).

We also screened for antimicrobial resistance genes in some selected isolates to understand the genetic basis for their antimicrobial resistance. The genes identified include tetracycline (*tetA*), fluoroquinolone (*gyrA*), sulfonamide (*sul1*), and ampicillin (*bla_TEM_*). The resistance to tetracycline was 68.6%, fluoroquinolone was 52.8%, sulfonamide 53.2%, and ampicillin 49.4%. The most common resistant antibiotic gene from this study was observed to be the tetracycline gene with 68.6%. The presence of these resistant genes has been recorded in previous studies (Momtaz et al., 2012; Dehkordi et al., 2014; Al Azad et al., 2019). Antimicrobial-resistant genes play a vital role in the spread of environmental resistance (Massé et al., 2014) especially in the food chain because food doubles as a vector for the transfer of residual antibiotics and for the transfer of antibiotic genes carried by zoonotic pathogens thus constituting public health and One Health concern. The misuse of antibiotics for prophylactic treatment and as growth promoters, poor biosecurity, and close clustering of different poultry in the same area are drivers for the emergence and spread of antimicrobial resistance. According to Manyi-Loh et al. (2018), commonly used antibiotics such as tetracyclines, aminoglycosides, β-lactams, lincosamides, macrolides, pleuromutilins, and sulfonamides by farmers worldwide can have negative effects on health. This raises concerns about disease control and risk management. We showed the intricate relationship between farm management practice and biosecurity.

Another important finding in this study is the presence of some virulence genes found in the isolates. One or more isolates were positive for each virulence gene tested. Of the 18 selected *E. coli*-positive isolates, 8 (44.44%) isolates contained the *eaeA gene*, and this is high when compared to similar data from two other studies on the *eaeA* gene in poultry conducted by Wani et al. (2004) and Enany et al., (2019), which recorded 2.49 and 20.3%, respectively. This is noteworthy because of the invaluable role the gene plays in the attachment and effacing of enteropathogenic *E. coli* (EPEC) in the intestinal mucosa (Rahimi et al., 2022); 1 (5.55%) out of the 18 isolates also contained the *hylA* gene, which is in line with the study by Wani et al. (2004), reporting a *hylA* prevalence of 1.49% of the *E. coli* isolates in chicken. These genes responsible for virulence and resistance in *Escherichia coli* have been identified in humans, especially poultry workers (Adelowo et al., 2014; Joshua et al., 2018), thus contributing to the spread of antimicrobial resistance. In their study, AMR is more frequent in pathogenic than in other commensal (non-pathogenic) *E. coli* strains. This suggests that the association between resistance and virulence genes is one that self-preserves the pathogen.

It has been projected that by 2050, almost 10 million people would die from bacterial infections that are resistant to antibiotics (O’Neill, 2019). Due to the threat of antibiotic resistance on health outcomes globally, the World Health Organization (WHO) considers antimicrobial resistance to be one of the major threats to public health as more bacteria are becoming resistant to more antibiotics (WHO, 2021). It is worrisome that most of these resistant bacteria are zoonotic, and infections triggered by these pathogens can be difficult to treat because previously potent drugs become less efficacious against the same pathogen. The resistance reported from this study may have emanated from lay farm owners, but it generally reflects poorly on a system of underperforming antimicrobial stewardship.

Although we set out to detect MDR and resistant genes in intensive and extensively managed poultry, our study was limited in terms of statistical power to make a compelling statement on MDR in local birds; the detection of resistant genes in local birds sampled indicates environmental contamination by resistant genes since all local birds sampled are not administered antibiotics compared to extensively managed birds. This study illuminates the complex interplay of farm practices, environmental conditions, and biosecurity measures in influencing the prevalence and spread of pathogenic and resistant bacteria. Notably, the significance of certain associations changes from univariate to multivariable models, highlighting the importance of considering a multifactorial approach to manage and mitigate bacterial risks on farms effectively.

## Conclusion

Antibiotics are of importance in agriculture, veterinary, and clinical settings. Notwithstanding the beneficial roles of antibiotics, their abuse may lead to serious public health complications. Results from this study indicate that there are significant levels of MDR and antibiotic resistance of *E. coli* in poultry farms, suggesting that there is a lot of antibiotic abuse in the poultry farming process. The presence of virulence genes associated with these isolates makes it more worrisome for public health, and this underscores the need for farmers to be educated on the appropriate use of antibiotics and stewardship.

## Data availability statement

The original contributions presented in the study are included in the article/Supplementary material, further inquiries can be directed to the corresponding authors.

## Ethics statement

The animal studies were approved by Animal ethics committee of the National Veterinary Research Institute NVRI, Vom. The studies were conducted in accordance with the local legislation and institutional requirements. Written informed consent was obtained from the owners for the participation of their animals in this study.

## Author contributions

EA: Writing – review & editing, Writing – original draft, Methodology, Investigation, Funding acquisition, Formal analysis, Data curation, Conceptualization. DK: Writing – review & editing, Writing – original draft, Methodology, Investigation, Formal analysis, Data curation, Conceptualization. NM: Writing – original draft, Methodology, Investigation, Conceptualization. RA: Writing – original draft, Methodology, Investigation, Conceptualization. CO-E: Writing – review & editing, Methodology, Investigation, Conceptualization. EB: Writing – review & editing, Methodology, Investigation, Conceptualization. JB: Writing – review & editing, Methodology, Investigation, Conceptualization. KS: Writing – review & editing, Methodology, Investigation. RR: Writing – review & editing, Methodology, Investigation, Conceptualization. GJ: Writing – review & editing, Methodology, Investigation, Conceptualization. BA: Writing – review & editing, Visualization, Validation, Supervision. GA: Writing – review & editing, Visualization, Validation, Supervision. OA: Methodology, Investigation, Formal analysis, Writing – review & editing, Visualization, Supervision. CM: Writing – review & editing, Visualization, Validation, Supervision.

## Supplementary material

SUPPLEMENTARY FIGURE S1 A Condition Typical of *Escherichia coli* Septicaemia as seen in one of the Farms.

SUPPLEMENTARY FIGURE S2 Gram Positive Short Rods Typical of *Escherichia coli* seen under Oil Immersion.

SUPPLEMENTARY FIGURE S3 Pathogenic isolates on Sorbitol Macconkey Agar Appear as yellow colonies and *Escherichia coli* on Eosin Methylene Blue Agar. The PCR reactions were performed in a final volume of 25 μl containing 7 μl nuclease free water, 12.5 μl 2x green master mix, 20 pmole of 0.25 μl of primer and 5 μl of DNA template. Tetracycline and quinolones had 0.25 μl of 20 pmole of each primer, while sulphonamide and ampicillin had 0.5 μl of each primer and 6.5 μl of nuclease free water respectively. PCR amplification cycle of Tetracycline resistant genes consisted of an initial denaturation temperature at 94 ^O^C for 5 min, followed by denaturation temperature at 94 ^O^C 1 min, annealing temperature at 55 ^O^C for 1 min and final elongation temperature at 72 ^O^C for 90 sec. PCR amplification cycle of quinolones resistant genes consisted of an initial denaturation temperature at 95 ^O^C for 5 min, followed by denaturation temperature at 94 ^O^C 1 min, annealing temperature at 56 ^O^C for 1 min and final elongation temperature at 72 ^O^C for 1 min. PCR amplification cycle of sulphonamide resistant genes consisted of an initial denaturation temperature at 94 ^O^C for 5 min, followed by denaturation temperature at 94 ^O^C for 1 min, annealing temperature at 55 ^O^C for 1 min and final elongation temperature at 72 ^O^C for 5 min. PCR amplification cycle of ampicillin resistant genes consisted of an initial denaturation temperature at 94 ^O^C for 5 min, followed by denaturation temperature at 94 ^O^C for 30 sec, annealing temperature at 50 ^O^C for 30 sec, and final elongation temperature at 72 ^O^C for 90 sec. Electrophoresis were carried out on the amplicons using 1.5% agarose powder in 100 ml of 1X TBE at 100v for 35 min. the gel were visualized using Gel Documentation System (Synegene®). A molecular weight marker with 100bp and 1kb plus was used as a size standard. An in-house positive control, extracted from *E. coli* as used by Biotechnology Centre of the National Veterinary Research Institute, Vom, Plateau State, Nigeria, was used as positive control, while reaction with no template DNA added was used as negative control.
