# Supplementary figures and images for: Prevalence of multidrug-resistant Escherichia coli isolates and virulence gene expression in poultry farms in Jos, Nigeria
Source: Front Microbiol. 2024 Jun 12;15:1298582. doi: 10.3389/fmicb.2024.1298582 (PMC11199394; doi:10.3389/fmicb.2024.1298582)

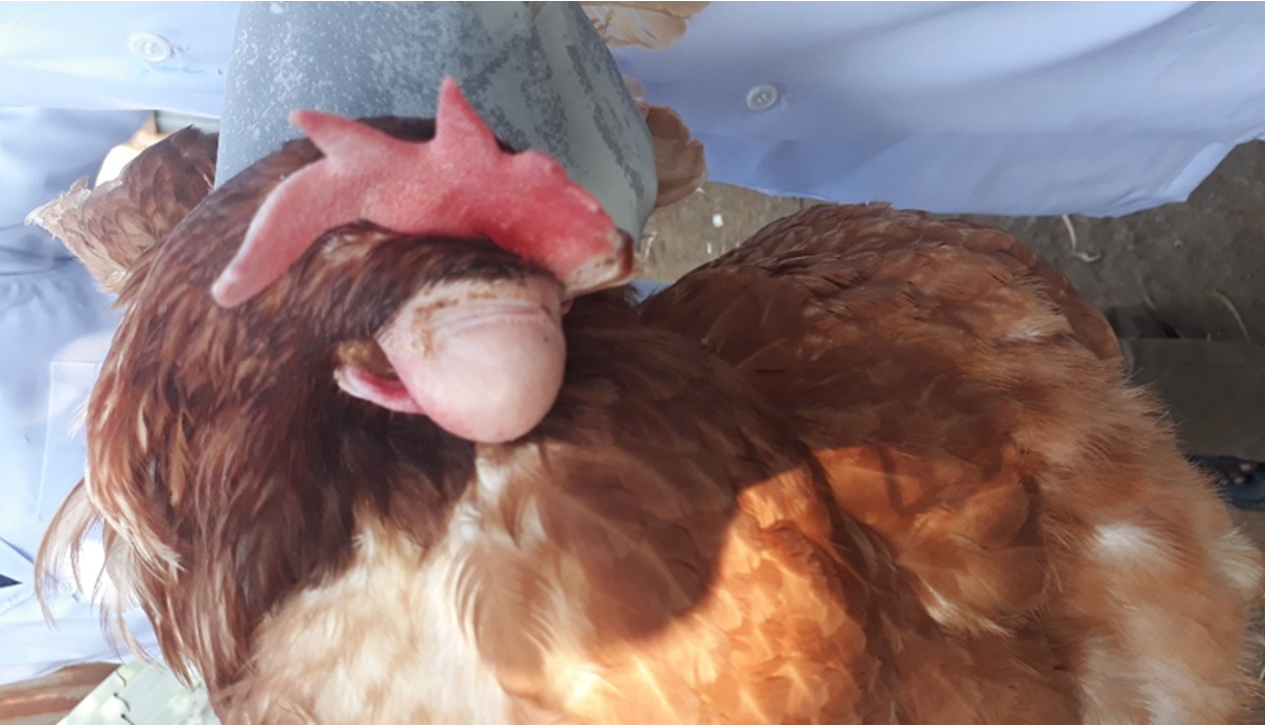

Supplement: SUPPLEMENTARY FIGURE S1 — A Condition Typical of Escherichia coli Septicaemia as seen in one of the Farms. [file Image_1.PNG]

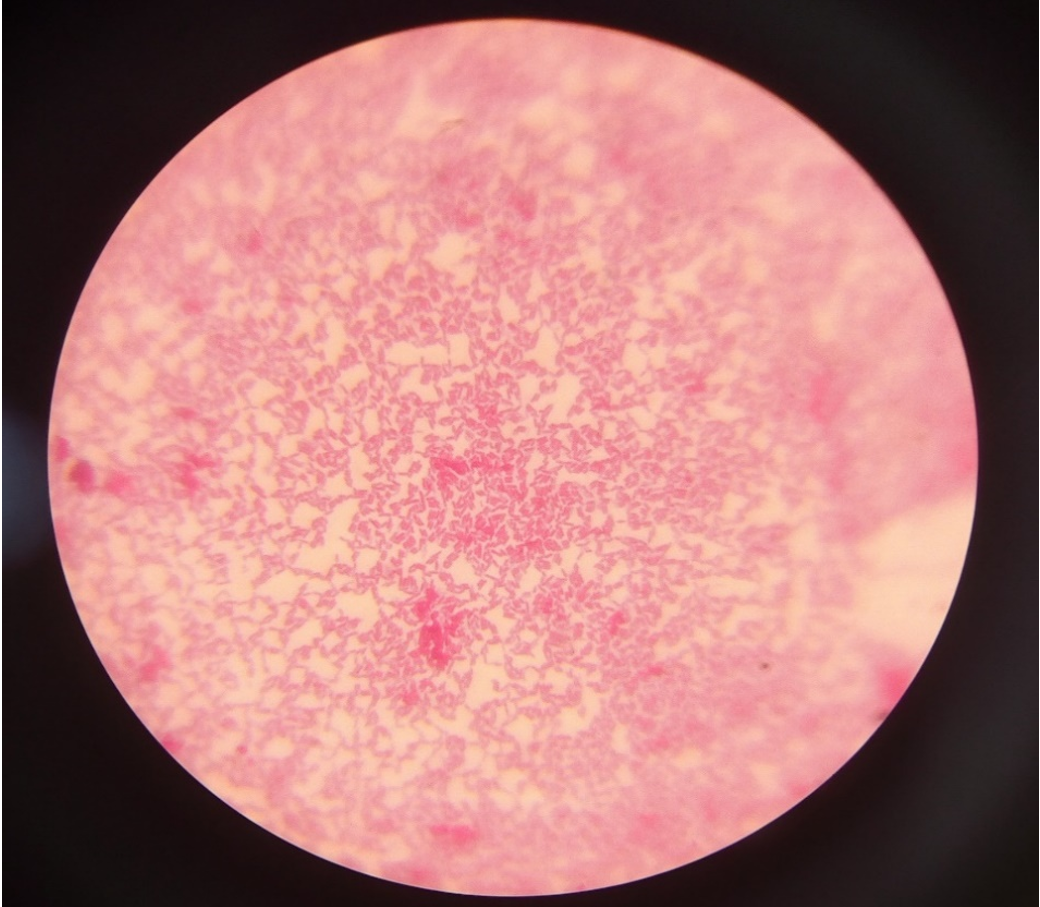

Supplement: SUPPLEMENTARY FIGURE S2 — Gram Positive Short Rods Typical of Escherichia coli seen under Oil Immersion. [file Image_2.PNG]

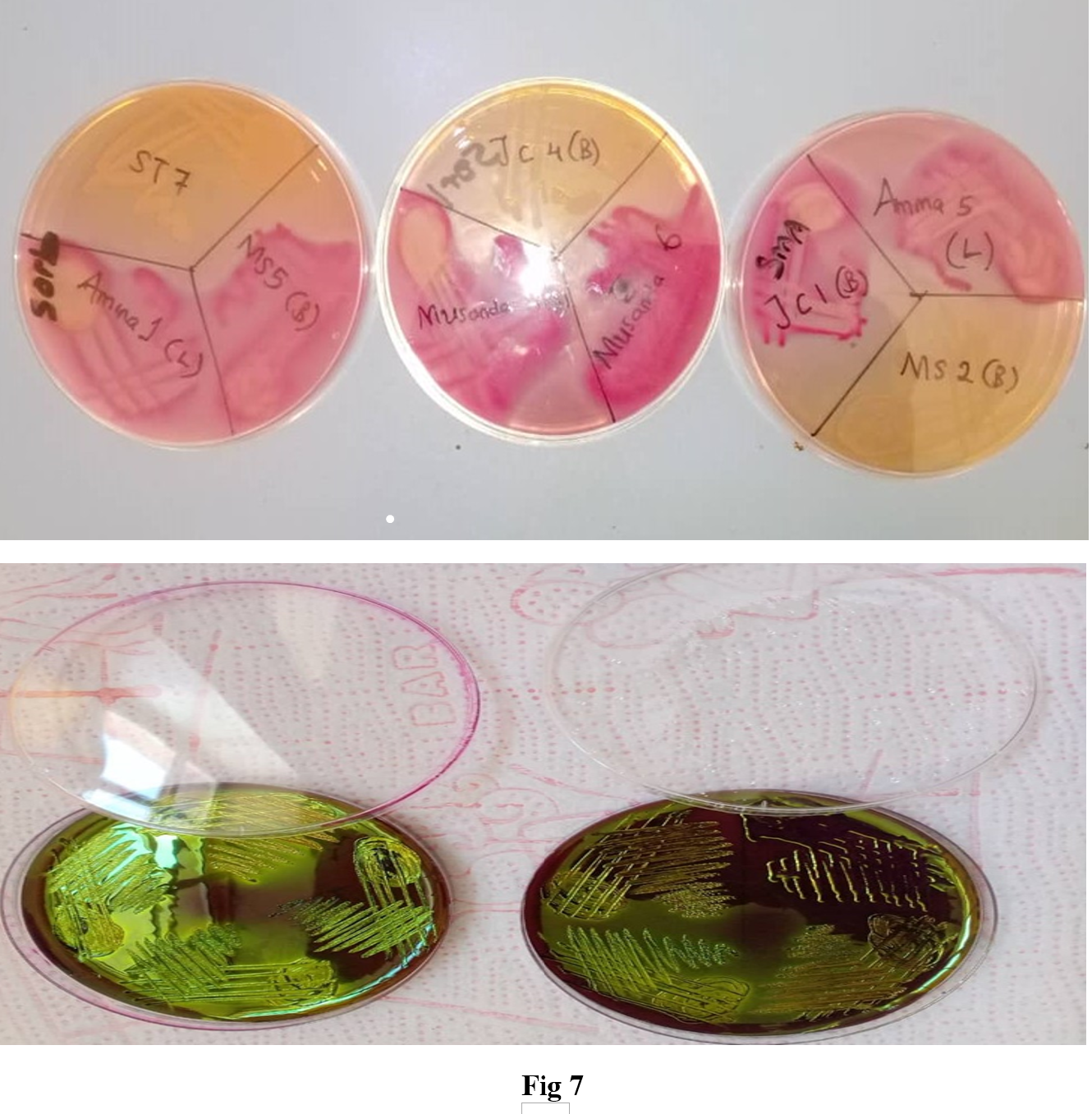

Supplement: SUPPLEMENTARY FIGURE S3 — Pathogenic isolates on Sorbitol Macconkey Agar Appear as yellow colonies and Escherichia coli on Eosin Methylene Blue Agar. The PCR reactions were performed in a final volume of 25 μl containing 7 μl nuclease free water, 12.5 μl 2x green master mix, 20 pmole of 0.25 μl of primer and 5 μl of DNA template. Tetracycline and quinolones had 0.25 μl of 20 pmole of each primer, while sulphonamide and ampicillin had 0.5 μl of each primer and 6.5 μl of nuclease free water respectively. PCR amplification cycle of Tetracycline resistant genes consisted of an initial denaturation temperature at 94 OC for 5 min, followed by denaturation temperature at 94 OC 1 min, annealing temperature at 55 OC for 1 min and final elongation temperature at 72 OC for 90 sec. PCR amplification cycle of quinolones resistant genes consisted of an initial denaturation temperature at 95 OC for 5 min, followed by denaturation temperature at 94 OC 1 min, annealing temperature at 56 OC for 1 min and final elongation temperature at 72 OC for 1 min. PCR amplification cycle of sulphonamide resistant genes consisted of an initial denaturation temperature at 94 OC for 5 min, followed by denaturation temperature at 94 OC for 1 min, annealing temperature at 55 OC for 1 min and final elongation temperature at 72 OC for 5 min. PCR amplification cycle of ampicillin resistant genes consisted of an initial denaturation temperature at 94 OC for 5 min, followed by denaturation temperature at 94 OC for 30 sec, annealing temperature at 50 OC for 30 sec, and final elongation temperature at 72 OC for 90 sec. Electrophoresis were carried out on the amplicons using 1.5% agarose powder in 100 ml of 1X TBE at 100v for 35 min. the gel were visualized using Gel Documentation System (Synegene®). A molecular weight marker with 100bp and 1kb plus was used as a size standard. An in-house positive control, extracted from E. coli as used by Biotechnology Centre of the National Veterinary Research Institute, Vom, Plateau State, Nigeri [file Image_3.PNG]
